# Serum levels of adipokines resistin and leptin in patients with colon cancer

**Published:** 2010-11-25

**Authors:** A Sălăgeanu, C ţucureanu, L Lerescu, I Caraş, R Pitica, G Gangura, R Costea, S Neagu

**Affiliations:** * Infection and Immunity Laboratory, National Institute for Research and Development in Microbiology and Immunology ‘Cantacuzino’ Romania; ** Department of Surgery, Emergency University HospitalRomania

**Keywords:** colon cancer, adipocytokine, resistin, leptin

## Abstract

Adipose tissue displays characteristics of an endocrine organ releasing a number of adipocyte–specific factors known as adipocytokines.It has been recently suggested that adipocytokines may play a role in pathogenesis and progression of certain cancers, in particular in colorectal cancer. The aim of this study was to investigate the association between several blood adipocytokine levels and clinicopathological characteristics of colon cancer patients undergoing surgery. The study group comprised of 29 patients who underwent surgical resection for colon cancer at Emergency University Hospital Bucharest and 27 healthy volunteers. The serum levels of adipocytokines were measured using multianalyte xMap profiling technology (Luminex). Resistin levels were significantly higher in colon cancer patients while leptin serum levels were significantly lower as compared to controls. Leptin levels decreased gradually with tumor stage and aggressiveness. Taken together, these results of this study suggest that adipokines, in particular resistin and leptin may be involved in development and progression of colon cancer.

## Introduction

In the past decade it has become increasingly clear that adipose tissue displays characteristics of an endocrine organ releasing a number of adipocyte–pecific factors known as adipocytokines [1,2]. It has been recently suggested that adipocytokines, such as TNF–alpha, IL–6, type–1 plasminogen activator inhibitor (PAI–1), adiponectin, leptin, resistin, visfatin and apelin are associated with the risk of cancer at various sites (e.g., breast, prostate gland, endometrium and colorectum) [3–6]. This might partially explained the epidemiological evidence of obesity–related carcinogenesis. The altered secretion of metabolically active, proinflammatory adipocytokines from adipose tissue is believed to play a key role in the mechanisms relating obesity and cancer which are only started to be uncovered [7]. Moreover, obesity is associated with a state of chronic low level inflammation, characterized by abnormal cytokine production which might affect both tumor initiation and tumor progression, such as adipocyte–conditioned medium can e.g. promote tumor migration [1,8]. 

Although a strong association between the prevalence of colorectal adenomas and increased levels of proinflammatory cytokines such as IL–6 and TNF–alpha has been documented [9] there are contradictory reports in the literature regarding the role of ‘classical’ adipocytokines such as leptin, adiponectin or resistin in this pathology. For example, in several studies, a significant increase in colon cancer risk with increasing serum leptin concentrations was reported [7,10] while others reported significantly lower leptin levels in cancer patients relative to controls [11]. Elevated levels of plasma resistin have been found in breast cancer [12] and in non–small cell lung cancer patients [13]. As it has been shown that resistin is produced by the stromovascular fraction of adipose tissue and peripheral blood monocytes, increased resistin levels in cancer patients was explained not only by the adipose tissue involvement but also by the activation of monocytes as part of the generalized inflammatory process. In one study, increased resistin and low adiponectin serum levels negatively correlated with the stage were reported in colorectal cancer [5]. Also, an upregulation in 92% of the samples in the levels of resistin protein in colon cancer tissue was observed [14]. Collectively, these studies suggest a possible role of adipokines in pathogenesis and progression of certain cancers, in particular of colorectal cancer.

The aim of this study was to investigate the association between several blood adipocytokine levels and clinicopathological characteristics of colon cancer patients undergoing surgery. 

## Materials and methods

### Study population

The study group comprised of 29 patients who underwent surgical resection for colon cancer at Emergency University Hospital Bucharest (Department of General Surgery, Division Ⅱ) and 27 healthy volunteers. All subjects (patients and controls) provided informed consent prior to the collection and analysis of blood samples and the study was approved by local Ethics Committee.

### Adipocytokines measurements

Blood samples were collected from colon cancer patients before and after surgery. Whole blood was allowed to clot for 1 hour at 37 degrees C and spun to serum (quick run). Serum was aliquoted and stored at –20 degrees C until analyzed. The following adipocykines were tested in serum samples: adiponectin, leptin, resistin and serpin/type–1 plasminogen activator inhibitor (PAI–1) using Human Obesity Multianalyte Profiling Base Kit and Fluorokine MAP Human microbeads for each of the above mentioned parameters (R and D Systems Inc., Minneapolis, USA) following the protocol described in detail elsewhere [15]. Plate reading was performed on the Luminex 200 platform (purchased from Luminex Corporation, Austin, TX, USA) and data were processed with Luminex 200 IS 2.3 Star Station software (Applied Cytometry, Plano, TX, USA).

### Statistical Analysis 

Statistical analysis was performed using R software [16]. Serum levels of individual adipocytokines between groups were compared by the nonparametric two–tailed Wilcoxon signed–rank test (unpaired for testing differences between groups and paired for the same group at different time points). Correlations between individual cytokine levels were analyzed by the nonparametric Spearman's correlation test. A P–value <0.05 were considered to be statistically significant.

## Results

Table 1 shows the clinical characteristics of the patients and the controls. There was no significant difference in age and sex between the 2 groups. 

**Table 1:** Clinical characteristics of patients and controls

Characteristic	Patients (n=31)	Controls (n=27)	P
Age (mean ±SD, range)	63 ± 13 (27–84)	59.1 ±14.8 (23–89)	0.21
Sex			
female	12	15	
male	17	12	
Tumor location			
ascending colon	7		
transverse colon	1		
descending colon	7		
sigmoid colon	14		
Tumor stage (Dukes')			
B1	2		
B2	17		
C	7		
D	1		
Not known	2		
Tumor differentiation			
Poorly differentiated	7		
Moderately diff.	15		
Well differentiated	2		
Mucinous	3		
Not known	2		

The adipokines concentration were measured in patients' serum at two time points: 3–4 days before surgery (time point 1: ‘preop’), and 5–7 days after surgery (time point 2: ‘postop’). The concentration of adiponectin and PAI–1 in undiluted serum was above the upper limit of linearity of the test for both patients and controls. Therefore, we decided to examine comparatively only the concentration of resistin and leptin which did not exceed the measurable range of the test in undiluted sera of patients and controls. 

**Figure 1:**
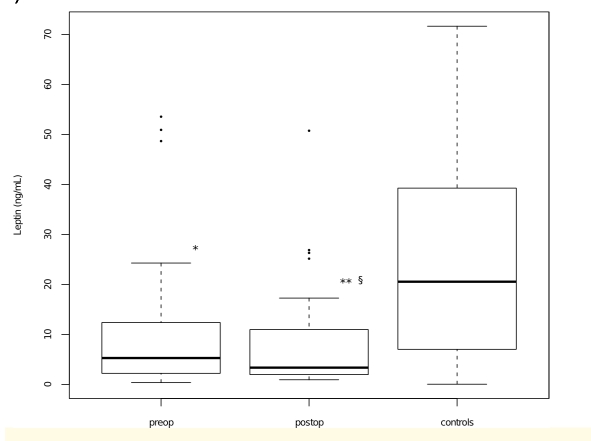
Box plots representing serum leptin levels in patients with colon cancer before surgery (preop) and after surgery (postop) and controls. (P < 0.05 vs controls; P < 0.01 vs controls; P < 0.01 vs preop, two–tailed Wilcoxon signed-rank test.

**Figure 2:**
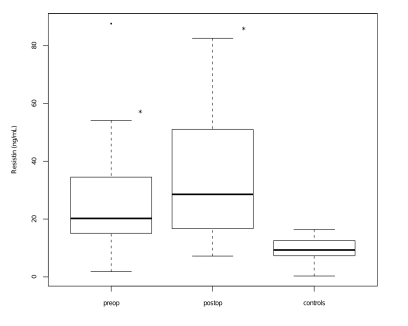
Box plots representing serum resistin levels in patients with colon cancer before surgery (Pre–op) and after surgery (Post–op) and controls. (P < 0.001 vs controls, two–tailed Wilcoxon signed-rank test)

As can be seen in (Figure 1), serum leptin levels measured both before and after surgery were significantly lower in patients compared to healthy controls (P=0.01 and P<0.001, respectively). Moreover, a statistically significant decrease of leptin concentration was noted after surgery as compared to presurgical levels (P<0.01). By contrast, resistin serum levels in colon cancer patients were significantly higher as compared to controls, both pre– and post operatively (P<0.001 in both cases) (Figure 2).  

Interestingly, for both mediators, we noticed a statistically significant positive correlation between preoperative and postoperative values (r=0.408, P<0.05 for resistin and r=0.701, P<0.001 for leptin). 

Next, we looked for possible association between measured adipokines serum levels and clinicopathological features such as tumor stage, histological grade of differentiation or localization.  There was no statistically significant difference in serum concentration of resistin or leptin between A+B vs. C+D Dukes' stage (Table 2). 

**Table 2:** Serum adipokines concentration and P value in colon cancer patients according to tumor stage (P<0.01, Dukes' B1+B2 carcinoma vs. controls, NS, Dukes' B1+B2 vs. Dukes' C+D,    P<0.001, Dukes' C+D vs. controls)

	Dukes' B1+B2 (n=19)		Dukes' C+D (n=8)	
	Mean ±SD	Med (Min, max)	Mean ±SD	Med (Min, max)
Resistin	27.25 ± 16.3	32.51 (6.13, 46.4)	27.45 ± 19.46	20.2 (1.84, 87.6)
Leptin	11.99 ±16.2	8.17 (1.33, 50.94)	12.62 ±15.8	5.3 (0.38, 53.6)

As can bee seen, for both subgroups of patients, serum resistin levels were significantly higher as compared to controls (P<0.01 for Dukes' B1+B2 stages and P<0.001 for Dukes' C+D stages). Leptin serum concentration was significantly decreased than those in controls only in patients in more advanced stages (P<0.05 as compared to controls). Moreover, a significant negative correlation was present between leptin and T stage (r=–0.218, P=0.021, Spearman); however no correlation was noticed between leptin concentration and Dukes' stage.  Disease stage and resistin displayed no correlation. 

Although a trend toward higher values was noticed for both tested mediators in patients with well and moderately differentiated tumors versus poorly differentiated or mucinous colon adenocarcinoma, the differences did not reach the statistical significance (Table 3). When compared control values, resistin levels were significantly higher in both patients' subgroups, while leptin levels were significantly lower only in the subgroup of patients with poorly differentiated (including the three patients with mucinous adenocarcinoma). 

**Table 3:** Serum adipokines concentration and P value in colon cancer patients according to tumor grade of differentiation (P<0.00001, Well/ Mod. differentiated carcinoma vs. controls; NS, Well/ Mod. differentiated carcinoma vs. Poorly differentiated + Mucinous carcinoma; P<0.01, Poorly differentiated + Mucinous carcinoma vs. controls)

	Well and moderately differentiated (n=17)		Poorly differentiated + Mucinous (n=10)	
	Mean ±SD	Med (Min, max)	Mean ±SD	Med (Min, max)
Resistin	30.91 ± 19.93	24.7 (6.13, 87.6)	21.41 ± 13.95	18.2 (1.84, 46.4)
Leptin	15.12 ±18.5	24.7 (0.71, 53.6)	7.86 ±7.6	5.31 (0.38, 23.4)

No significant differences in adipokines serum concentration according to tumor localization were noticed (P>0.05). 

## Discussion

The aim of this study was to determine the correlation, if any, between clinicopathological features of patients with colon cancer undergoing surgery and the serum level of adipokines. 

We observed that circulating resistin levels were significantly increased in colon cancer patients as compared to controls. These results are in agreement with other studies which found higher levels of resistin in patients with colorectal [5,11], breast [6] or non–small cell lung cancer [13]. Nakajima et al. reported that resistin levels were significantly higher in colorectal cancer patients than those in controls independent of the BMI, and these levels gradually increased with progression in tumor stage [17]. In our study, although colon cancer patients displayed increased serum resistin levels, we did not obtain any correlation between serum levels of resistin and tumor stage, localization or grade of differentiation. This discrepancy might be explained by the limited number of patients in advanced stage of disease (n = 8 in Dukes' C+D stages). 

It has been suggested that high resistin levels are related to cancer associated chronic inflammation. Recent data indicate that stimulation of macrophages in vitro with endotoxin or proinflammatory cytokines leads to a marked increase in resistin production and vice versa, resistin strongly up–regulate IL–6 and TNF–alpha production [6,18]. Thus resistin seems to act as an important member of the cytokine family with potent regulatory functions [18]. In preliminary studies we found significantly higher levels of both pro–inflammatory cytokines (TNF–alpha, IL–6, IL–8) and anti–inflammatory cytokine IL–10 in colon cancer patients as compared to healthy subjects (unpublished observations). However, in our study, there was no correlation between these cytokines and resistin levels, neither before, nor after surgery (data not shown). Collectively, our results showed a statistically significant increase in resistin serum levels but a lack of direct involvement of resistin in the systemic inflammatory response of patients with colon cancer or in disease progression.

The association between leptin levels and the risk of colorectal cancer or adenoma has remained controversial. Although it has been suggested that in men, leptin may be associated with risk of colorectal adenomas [10,19] other studies found decreased leptin serum levels in colorectal cancer patients [11]. We found significantly lower serum leptin levels in colon cancer patients as compared to controls. Moreover, a significant negative correlation between leptin and T stage (P=0.021) was noted. When patients were divided in two subgroups according to tumor grade of differentiation, we found statistically significant lower serum leptin levels only for patients with poorly differentiated tumors and mucinous adenocarcinoma, additionally suggesting a gradually decrease in leptin levels with tumor aggressiveness. Others did not observe any significant differences for the fasting serum leptin levels in patients according to pTNM staging although a role for the leptin receptor is discussed in relation with the tumor immune response [20]. A plausible explanation for the inconsistencies in results includes differences in techniques for leptin detection, timing of phlebotomy and duration of fasting. 

Our study has some limitations. We had no information regarding body weight changes in the patients and controls before the sampling, and thus it was not possible to determine whether the changes in adipokines levels in the patients was caused by obesity before the sampling. Notably, several studies made the observations that adipokines concentration was not correlated with anthropometric measures of adiposity or weight loss in both control subject and cancer patients [6,11]. However, other studies confirm a strong correlation between leptin level and body mass index [20]. Also, if many more patients were enrolled in this study, significant correlation between adipokines levels and clinicopathological features may have been detected. Despite these limitations we demonstrated that the levels of resistin and leptin were significantly different between colon cancer patients and controls. Resistin had significantly higher serum values in patients while leptin serum levels were lower as compared to controls. Leptin levels decreased gradually with tumor stage and aggressiveness. Moreover, for both adipocytokines, a statistically significant positive correlation between preoperative and postoperative values was noted. Taken together, these results of this study suggest that adipokines, in particular resistin and leptin might be involved in development and progression of colon cancer.  Obviously, further studies are needed to better elucidate which role adipocytokines have in determining disease progression. 
